# Multiple antiarrhythmic transplacental treatments for fetal supraventricular tachyarrhythmia: A protocol for systematic review and meta analysis

**DOI:** 10.1097/MD.0000000000023534

**Published:** 2020-12-11

**Authors:** Tingting Chen, Yanfeng Yang, Kun Shi, Yue Pan, Sumei Wei, Zexuan Yang, Xiao Yang

**Affiliations:** a Department of Pediatric Cardiology; b Department of Obstetrics; c Department of Ultrasound, Chengdu Women's and Children’ s Central Hospital, School of Medicine, University of Electronic Science and Technology of China, Chengdu, China.

**Keywords:** atrial flutter, fetal tachycardia, meta-analysis, supraventricular tachycardia, systematic review, treatment

## Abstract

**Background:**

Fetal supraventricular tachyarrhythmia is a common reason for referral to fetal cardiology. Multiple antiarrhythmic transplacental medications can be used to treat these diseases. Debates remain regarding the standardized therapy.

**Methods:**

PubMed, EMBASE, Cochrane Library, Web of Science, Google Scholar, and ClinicalTrials.gov will be searched from inception to September 2020. A handsearching for gray literature, including unpublished conference articles, will be performed. The randomized control trials, case–control, and cohort studies will be accepted, no matter what the languages they were reported. We will first focus on the effectiveness of the therapy on fetal cardiac rhythm and/or heart rate. Then we will do further analysis of preterm delivery, fetal hydrops, intrauterine fetal demise, and maternal side effects. The Cochrane Risk of Bias Tool and the Newcastle–Ottawa scale will be used to assess the risk of bias of the randomized controlled trials, case–control, and cohort studies, respectively. Two independent reviewers will carry out literature identification, data collection, and study quality assessment. Discrepancies will be resolved by a third reviewer. Statistical analysis will be conducted using the STATA 13.0 software.

**Result:**

The results will provide helpful information about the effect of multiple antiarrhythmic transplacental therapies in pregnancies with supraventricular tachycardia or atrial flutter, and demonstrate which therapy is more effective.

**Conclusion:**

The conclusion drawn from this systematic review will benefit the patients with fetal supraventricular tachyarrhythmia.

## 1 Introduction

Fetal tachyarrhythmia is cardiac rhythms and/or heart rates >180 beats per minute, which develops in <0.1% of pregnancies.^[1,2]^ The most common forms of fetal tachyarrhythmia include supraventricular tachycardia (SVT) and atrial flutter (AF).^[3,4]^ Sustained SVT is defined as any of the above nonsinus tachyarrhythmias that present for ≥50% of the fetal scan time, which usually occurs at rates ≥220 bpm, includes atrioventricular reentrant tachycardia, persistent junctional reciprocating tachycardia, congenital junctional ectopic tachycardia, and ectopic atrial tachycardia. When persistent, tachyarrhythmia can lead to fetal nonimmune hydrops, cardiac dysfunction, premature delivery, or even fetal demise.^[5,6]^

Fetal rhythm conversion to sinus rhythm or rate control via antiarrhythmic transplacental therapy was reported 40 years ago.^[7]^ Multiple studies have found digoxin, flecainide, sotalol, amiodarone, and other antiarrhythmic agents to be effective in the treatment of fetal SVT and AF.^[8]^ Digoxin is often the first-line agent for management of fetal SVT without hydrops fetalis because of its long history of use during pregnancy, and clinician familiarity with its use.^[9–12]^ Compared with digoxin, flecanide, and sotalol cross the placental barrier easier, especially in hydropic fetuses. Therefore, some researchers proposed sotalol or flecainide as first-line agent.^[12–15]^ As more significant toxicity profile for the expectant mother and fetus than other drugs, amiodarone is reserved as second-line or third-line therapy of drug-refractory tachycardia with hydrops and/or cardiac dysfunction.^[16,17]^ Combination treatments (digoxin/flecainide, digoxin/sotalol, amiodarone/digoxin, amiodarone/flecainide, and sotalol/flecainide) have been reported to be effective when other agents have failed.

There is no standardized treatment of fetal supraventricular tachyarrhythmia. The choice of antiarrhythmic therapy is controversial. Alsaied et al^[8]^ conducted on the efficacy, safety, and fetal–maternal tolerance of first-line monotherapy for fetal SVT and AF. Unfortunately, effective treatment requires multiple antiarrhythmic agents in more than 50% of patients.^[18]^ However, there is currently no systematic review and meta-analysis of multiple antiarrhythmic transplacental treatments. The primary aim of this study is to undertake a comprehensive systematic review and meta-analysis to evaluate the polytherapy for fetal supraventricular tachyarrhythmia, and tease out what factors influence their effectiveness.

## 2 Methods

### 2.1 Registration

This study protocol has been registered on the International Platform of Registered Systematic Review and Meta-analysis Protocols (INPLASY), and the registration number is INPLASY2020100063. The Cochrane Handbook for Systematic Reviews of Interventions is used as a guideline.^[19]^ This protocol is conducted according to the Preferred Reporting Items for Systematic Reviews and Meta-Analysis Protocol.^[20]^

### 2.2 Criteria for including studies

#### 2.2.1 Types of studies

This article will review comparative original studies (the randomized control trials, case-control, and cohort studies) that made a comparison between different combination medication options as treatment of fetal supraventricular tachyarrhythmia, without any restrictions on language, date of transmission, or type of publication. Case reports, single-arm, and cross-section studies will be excluded.

Fetal supraventricular tachyarrhythmia includes supraventricular tachycardia (SVT) and atrial flutter (AF).

#### 2.2.2 Types of participants

Patients with sustained SVT, or AF, or patients with nonsustained fetal SVT with evidence of cardiac dysfunction and/or hydrops, who are treated with multiple **a**ntiarrhythmic transplacental therapy will be included in the analysis, regardless of their ethnicity and background. Patients with isolated premature atrial contractions and complete heart block were excluded.

#### 2.2.3 Types of interventions

We define multiple antiarrhythmic transplacental treatments as the experimental intervention, which is defined as digoxin, amiodarone, flecainide, or sotalol polytherapy. The efficacy of different combinations of these drugs will be compared.

#### 2.2.4 Types of outcome measures

The primary outcome will be the effect of therapy, defined as fetal rhythm conversion to sinus rhythm (SR) or rate control in those fetuses suffering SVT or AF who received multiple antiarrhythmic transplacental treatments.

The secondary outcomes will consider preterm delivery (<37 weeks’ gestation), fetal hydrops, intrauterine fetal demise, and maternal side effects.

### 2.3 Search strategy

We will search electronic databases including PubMed, EMBASE, Cochrane Library, Web of Science, Google Scholar, and ClinicalTrials.gov from inception to September 2020. The reference lists of studies and relevant systematic reviews for additional trials will be also scanned. For a comprehensive search, a search strategy that combines MeSH terms and free words will be adopted. Search strategy in PubMed is shown in Table 1.

**Table 1:** Search strategy for PubMed.

Number	Search terms
1	Fetus [MeSH]
2	(fetus OR fetal OR foetus):[tiab]
3	1 OR 2
4	Tachycardia, Supraventricular [MeSH]
5	(supraventricular tachyarrhythmia OR supraventricular tachycardia OR atrial flutter):[tiab]
6	4 OR 5
7	Therapeutics [MeSH]
8	(therapy OR treatment OR cure OR remedy OR antiarrhythmic transplacental treatment OR intrauterine treatment):[tiab]
9	Medicine[MeSH]
10	(medicine OR medication OR medicant): [tiab]
11	Drug [MeSH]
12	(drug OR transplacental antiarrhythmic drug): [tiab]
13	Anti-Arrhythmia Agents [MeSH]
14	(antiarrhythmic OR digoxin OR amiodarone OR flecainide OR sotalol): [tiab]
15	7 OR 8 OR 9 OR 10 OR 11 OR 12 OR 13 OR 14
16	3 AND 6 AND 15

### 2.4 Data collection and extraction

#### 2.4.1 Study Selection

**Figure 1:**
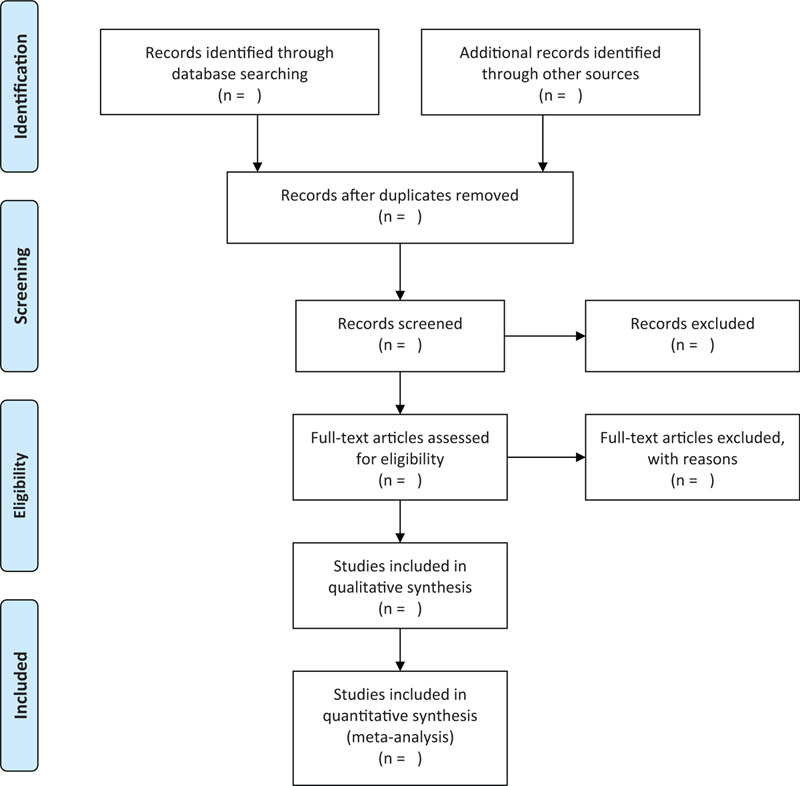
Flow chart of study flow from systematic search to selection process.

We will export the identified records in databases into EndNote X7 software. After removing duplicates, 2 reviewers will independently carry out study selection according to the previously inclusion criteria based on the title and abstract. Full-texts of relevant studies which are potentially eligible will be obtained to check for further evaluation. Any different opinions between 2 authors should be discussed and made an agreement with the third author. A flow diagram for the selection process will be developed using the PRISMA guidelines (Fig. 1).^[21]^

#### 2.4.2 Data extraction

Two authors will extract the data needed and fill out the data extraction form independently. The contents will include: generation information of the study (author, year of publication, region, among others), population (number, baseline characteristics, diagnosis, among others), study characteristics (design, randomization method, blinding, among others), intervention (drugs, administration, duration, among others), outcomes (conversion to SR, rate control, preterm delivery, among others). When the data of articles are insufficient or ambiguous, we will contact with the original author to request more essential information about the research by e-mail. Any discrepancies will be discussed and resolved in discussion with a third author.

### 2.5 Risk of bias assessment

Different methodological quality assessment tools will be used according to the types of the included studies by 2 assessors independently. Following the guidance in the latest version of Cochrane Handbook for systematic reviews of interventions,^[22]^ the version 2 of the Cochrane risk-of bias tool for randomized trials (RoB 2) will be used to assess the risk of bias for randomized clinical trials.^[23]^ The Newcastle–Ottawa scale^[24]^ will be used to assess the risk of bias of case–control studies, and cohort studies. The third reviewer will arbitrate in the case of any disagreement. Studies with high risk of bias or unclear bias will be given less weight in our data synthesis.

### 2.6 Data synthesis and statistical analysis

#### 2.6.1 Data synthesis and Assessment of heterogeneity

Statistical analyses will be performed using STATA software (version 13; StataCorp, College Station, TX). The dichotomous endpoints will be pooled and described as the odds ratio (OR) with 95% confidence interval (95% CI). For continuous data, the mean difference (MD) will be used if outcomes were measured in the same way between trials. The results that measured the same outcome but used different methods will be expressed as standardized mean difference (SMD) along with its 95% CI. Data that cannot be synthesized will be described qualitatively. Heterogeneity will be assessed by the*χ*^2^ test and *I*^2^ statistics. If *P* < .10 and *I*^2^ > 50%, the heterogeneity is significant and the random-effect model will be used for data analysis; otherwise, the fixed-effects model will be used. Funnel plot and Egger test will be applied to detect the potential reporting biases if >10 studies are included.

#### 2.6.2 Sensitivity analysis and subgroup analysis

To check the robustness of pooled outcome results, sensitivity analysis will be conducted based on the type of study, the missing data result, and the methodological quality of the included studies, when studies are adequate. We will repeat the analysis after excluding trials with a high risk of bias. If appropriate data are available, subgroup analyses will be exploratory according to the research design, therapy, and different outcomes.

#### 2.6.3 Confidence in cumulative evidence

The quality of evidence for all outcomes will be assessed by the Grading of Recommendations Assessment, Development, and Evaluation (GRADE) system. High, medium, low, or very low represents the 4 levels of evaluation.

## 3 Discussion

Untreated, sustained SVT and AF are associated with significant morbidity and mortality.^[25,26]^ Prenatal treatment is warranted for improving the fetal survival rate. However, in the treatment of fetal tachyarrhythmia, there is no consensus on the regimen of drugs. Most of the available literature suggests initial treatment with digoxin, or sotalol, or flecainide alone. Two systematic reviews recommended flecainide as first-line treatment for fetal SVT. But at the same time, both studies showed the lack of prospective studies as a limitation.^[8,27]^ To convert to SR and normal fetal heart rate, a portion of patients need to be treated with multiple medications. Therefore, it is necessary to perform a systematic review and meta-analysis to investigate the effects of multiple antiarrhythmic transplacental treatments on pregnancies with fetal supraventricular tachyarrhythmia which therapy is more effective. This study will be helpful to doctors treating fetal supraventricular tachyarrhythmia and provide some useful information when they are making the choice on which kind of medicine to be used.

## Author contributions

**Conceptualization:** Tingting Chen.

**Data curation:** Tingting Chen, Yanfeng Yang.

**Formal analysis:** Tingting Chen, Yanfeng Yang, Kun Shi.

**Funding acquisition:** Xiao Yang.

**Methodology:** Tingting Chen, Kun Shi, Yue Pan.

**Software:** Tingting Chen, Sumei Wei, Zexuan Yang.

**Supervision:** Xiao Yang.

**Writing – original draft:** Tingting Chen.

**Writing – review & editing:** Xiao Yang.
